# Knowledge and self-efficacy in starting advance care planning among community health and social care professionals in macao: A cross-sectional study

**DOI:** 10.1016/j.ijnsa.2026.100716

**Published:** 2026-10-18

**Authors:** Kuai In Tam, Sok Leng Che, Sok Man Leong

**Affiliations:** Complexo de Cuidados de Saúde das Ilhas – Edifício do Instituto de Enfermagem Kiang Wu de Macau, Avenida do Hospital das Ilhas no.447, Coloane, RAEM, Macao Special Administrative Region, China

**Keywords:** Advance care planning, Self-efficacy, ACP knowledge, Long-term care, Health and social care professionals

## Abstract

**Background:**

Advance care planning (ACP) has limited uptake in Macao despite increasing public interest. Little is known about frontline professionals’ readiness to initiate ACP in community and long‑term care.

**Aims:**

To assess ACP knowledge, self‑efficacy, and practice among community‑based health and social care professionals in Macao, and to examine how these factors relate to ACP implementation.

**Design:**

Cross‑sectional survey of physicians, nurses, therapists and social workers working across tertiary hospitals, community day centres and community long‑term care facilities in Macao.

**Methods:**

Structured questionnaire covering demographics, ACP knowledge (11 true/false items), self-efficacy in ACP communication (11 items, 1–10 scale), and ACP practice engagement (four scored items coded 0–2 and summed to 0–8, plus two unscored conditional follow-up questions). Descriptive statistics, correlations and regression explored associations between knowledge, self-efficacy and practice engagement.

**Results:**

A total of 213 professionals participated (nurses 67.1%; physicians 6.6%; therapists 7.5%; social workers 18.8%). Engagement in ACP activities was generally low, with 14.1% reporting prior ACP discussions with patients or families. ACP practice engagement was positively associated with knowledge (r = 0.25, p < 0.001) and self-efficacy (r = 0.33, p < 0.001). In the final regression model, self-efficacy had the largest standardised coefficient (β=0.25, p < 0.001); knowledge, master’s-level education, organisational ACP implementation, and work setting were also independently associated with practice engagement. Prior ACP training was significant in Model 1 but not in the final model (B = 0.33, p = 0.24). The final model explained 25.5% of the variance in ACP practice engagement.

**Conclusions:**

Advance care planning practice among health and social care professionals in Macao remains limited. Although both ACP knowledge and self-efficacy were associated with ACP practice, self-efficacy emerged as the stronger predictor of professionals’ engagement in ACP discussions.

**Implications:**

Improving ACP uptake in Macao requires skills-based training that combine knowledge acquisition with experiential communication to strengthen professionals’ self-efficacy. Organisational support and structured ACP implementation processes are also needed to facilitate sustainable ACP practice.

**What is known about this topic**

• Advance care planning improves alignment of care with patient preferences but is under‑used in many Asian settings.
• Professionals frequently report barriers around skills and confidence for advance care planning conversations.

**What this paper adds**

• Health and social care professionals in Macao show limited engagement in ACP despite moderate knowledge levels and generally positive willingness to participate in future ACP discussions.
• Both ACP knowledge and self-efficacy were associated with ACP practice, with self-efficacy emerging as the stronger predictor of professionals’ engagement in ACP.
• Strengthening ACP implementation in Macao requires experiential communication training to build professional self-efficacy, supported by organisational implementation and enabling policy contexts.

## Introduction

Advance care planning (ACP) refers to the process of communication whereby individuals, in discussion with healthcare providers and family, clarify their values and preferences for future care, particularly for end-of-life situations (Fromme et al., 2023; Rietjens et al., 2017; van der steen et al., 2024). In Western countries, ACP and advance directives (ADs) have been part of healthcare practice for decades, for example, the United States’ Patient Self-Determination Act of 1990 (United States Congress, 1990) and the United Kingdom’s Mental Capacity Act of 2005 (The Mental Capacity Act 2005) legally support individuals’ rights to document care decisions in advance. In many Asian regions, ACP is a relatively newer concept, but progress has been made: Singapore (Singapore, 1996) and Taiwan (Taiwan Ministry of Health and Welfare, 2015) enacted legislation to promote ADs and ACP discussions, and Hong Kong began encouraging ACP conversations over a decade ago (Hospital Authority, 2025). By contrast, Macao currently has no official policy or legislation on ADs or ACP. Public initiatives or guidelines on ACP is minimal, reflecting a significant gap in end-of-life care planning in the territory (The Macao SAR Government, 2016).

Despite the lack of formal ACP programmes, there is evidence of public interest in ACP in Macao. A local study found that 85.5% of respondents would like to discuss their future care plans and treatment preferences with healthcare professionals at the end of life (Leong et al., 2021). Recent local work has also reported gaps between expectations and actual ACP practice in long-term care and validated an ACP Practice Scale for health and social care professionals (Che et al., 2024; Tam et al., 2024). Official statistics indicate that adults aged 65 years or older comprised 14.6% of Macao’s population in 2024 (Statistics and Census Service, 2025). An earlier survey in China reported that almost 80% of adults had never heard of ACP, yet the majority wished to be informed of their medical condition and make their own healthcare decisions when facing serious illness (Zhang et al., 2015). More recent Mainland China’s studies have reported continuing gaps between favourable attitudes and routine clinical practice and moderate ACP acceptance among older adults (Cai et al., 2023; Yap et al., 2025). These findings highlight both limited awareness of ACP and a strong underlying desire for autonomy and discussion, suggesting unmet needs that ACP could address.

Health and social care professionals (HSCPs) have a pivotal responsibility in initiating ACP conversations with patients and families to honour their preferences. Proactively engaging patients in ACP can ensure that care decisions align with the person’s values and can reduce stress and anxiety for family members by avoiding last-minute crises (Gilissen et al., 2017). When ACP is done effectively, families experience less decisional burden and are better prepared, allowing them to focus on spending quality time with their loved one rather than agonising over choices during end-of-life situations (Gilissen et al., 2018, 2021). Achieving such benefits requires that HSCPs integrate ACP discussions as a routine part of care for those with serious or advanced conditions.

However, numerous studies indicate that professionals often struggle to implement ACP in practice (Rahemi et al., 2023; Zhang et al., 2021; Zhu et al., 023). Even in hospital settings with high acuity, nurses and other clinicians have reported low involvement in ACP discussions (Martina et al., 2021; Zwakman et al., 2018). Identified barriers are multifaceted. Lack of formal education or training in how to conduct end-of-life conversations is a major factor (Goto et al., 2022; Tan et al., 2024). In one study, over half of nursing staff had little to no training in ACP, which corresponded with uncertainty and discomfort in addressing the topic (Gilissen et al., 2020). Moreover, a prevailing culture in some healthcare settings tends to prioritise curative treatments and may avoid discussing death, making ACP feel countercultural or unsupported (Martina et al., 2021). Recent evidence also identifies unclear professional roles, limited time, fragmented documentation, and insufficient organisational support as implementation barriers (Inokuchi et al., 2023; Suen et al., 2024; Zhu et al., 2023).

A key barrier frequently identified is HSCPs’ lack of confidence in initiating ACP discussions (Chan et al., 2020; Suen et al., 2024; 2Zhu et al ., 2023). Even when professionals possess basic knowledge about ACP, they may not feel emotionally and skilfully equipped to handle what can be complex, emotional conversations. A recent review noted that insufficient confidence and commitment among HSCPs impeded ACP engagement (Martina et al., 2021). This lack of self-efficacy can result in missed opportunities to discuss patients’ preferences.

This relationship between knowledge, confidence, and behaviour can be understood through Bandura’s social cognitive theory, which proposes that self-efficacy, an individual’s belief in their capability to successfully perform a specific behaviour, is a key determinant of whether knowledge is translated into action (Bandura, 1977; Bandura and Wessels, 1997). Individuals who possess adequate knowledge may still avoid performing a task if they lack confidence in their ability to do so effectively. Within the context of ACP, initiating conversations about serious illness, future care, and end-of-life decisions requires not only factual knowledge but also confidence in communication, emotional management, and navigating family dynamics. Therefore, self-efficacy may represent a more proximal determinant of ACP practice than knowledge alone, providing a theoretical basis for examining both constructs in the present study.

Macao’s situation reflects these global challenges and adds local cultural considerations. In traditional Chinese culture, medical decisions for an incapacitated or seriously ill person are often made collectively by the family, rather than by the individual alone (Cheung et al., 2020; Fan, 1997). Family members typically play a significant role in end-of-life decision-making for older adults. Consequently, effective ACP in Macao must involve not only patients but also their relatives. Indeed, studies in Hong Kong have found that family members frequently look to HSCPs for guidance on end-of-life decisions, and such professional advice can strongly influence whether families support completing ADs (Cheung et al., 2020). Although the Macao SAR’s government has recognised population ageing as a major policy priority through the Ten-Year Action Plan for Elderly Services (The Macao SAR Government, 2016), ACP has not yet been formally incorporated into healthcare policy or supported by a legislative framework. Consequently, HSCPs are required to navigate culturally sensitive end-of-life discussions without clear institutional guidance or legal protection. Within a society where filial responsibility, collective family decision-making, and taboos surrounding death remain influential, this combination of cultural expectations and limited policy support may further undermine professionals’ confidence in initiating ACP conversations. If HSCPs in Macao are not prepared or proactive in discussing ACP, families may lack the information and encouragement needed to engage in planning. The success of ACP in this cultural context relies heavily on professionals initiating and facilitating conversations among patients and family members.

Although ACP has been investigated internationally, existing studies have predominantly focused on physicians or nurses working in acute hospital settings, and most evidence originates from Western healthcare systems where ACP policies are relatively well established. Evidence examining interdisciplinary HSCPs working in community settings within Chinese societies remains scarce. This represents an important gap because community-based professionals are increasingly expected to initiate ACP conversations early in the disease trajectory, yet their preparedness may be influenced by distinct cultural, organisational, and policy contexts. Recent reviews of ACP in nursing homes and dyadic interventions likewise support coordinated approaches involving professionals, patients or residents, and families (Burks et al., 2023; Liu et al., 2024).

Recognising these gaps, the present study is a pioneering effort to explore ACP implementation in Macao. In particular, Macao provides a unique context because of its rapidly ageing population, strong family-oriented cultural values, and the absence for informing future education, service development, and policy initiatives. Guided by Bandura’s concept of self-efficacy, this study examined both HSCPs’ ACP knowledge and their confidence in initiating ACP discussions, recognising that knowledge alone may be insufficient to facilitate behaviour change. Specifically, we conducted a cross-sectional survey to establish baseline data on (1) HSCPs’ ACP knowledge, (2) self-efficacy in initiating ACP discussions among HSCPs and (3) their current ACP practices. By examining the relationships between knowledge, self-efficacy, and practice, we aimed to identify which factors most strongly enable or impede ACP engagement by HSCPs.

## Methods

### Study design and setting

The study conducted a descriptive cross-sectional survey. Macao has a rapidly aging population (Documentation and Information Centre of the Statistics and Census Service, 2023), and like many regions, is challenged to improve end-of-life care. At the time of the study, there were no established ACP programmes or formal protocols in these facilities, making it necessary to assess baseline ACP-related knowledge and behaviours of HSCPs in Macao. The survey was conducted between December 2022 and May 2023.

### Participants and sampling

The study recruited HSCPs working in tertiary hospitals, community centres for older adults, and long-term care facilities in Macao. We included professionals of any age or years of experience, provided they had direct roles in caring for patients. We aimed to capture a multidisciplinary perspective since ACP discussions often involve a team.

Participants were recruited using convenience sampling.

A post hoc power analysis was conducted using G*Power 3.1 (Faul et al., 2007). Based on a multiple linear regression model with eight predictors, a medium effect size (f² = 0.15), an α level of 0.05, and a total sample size of 213, the achieved statistical power was 0.99, indicating that the study was adequately powered.

### Measures

The questionnaire was adapted from previously published instruments assessing ACP knowledge, self-efficacy, and practice. Specifically, the knowledge items were adapted from Chen et al. (2019), the practice items from Chen et al. (2019) and Hsieh et al. (2019), and the self-efficacy items from Chen et al. (2017). Similar psychometric properties have subsequently been demonstrated in the validated Chinese ACP self-efficacy scale (Yankg et al., 2022). The structured questionnaire comprised four domains: (1) socio-demographic characteristics (12 items); (2) knowledge of ACP (11 true/false statements); (3) self-efficacy in initiating ACP conversations (11 items, rated from 1 = not at all confident to 10 = completely confident); and (4) ACP practice, comprising four scored items with three response options and two conditional follow-up questions about the number and documentation of ACP discussions. In total, the instrument comprised 40 items.

Content validity was established during instrument adaptation through evaluation by a panel of three palliative and end-of-life care experts (two from Macao, one from Beijing). They evaluated semantic, idiomatic, experiential, and conceptual equivalence of the items. The content validity index (CVI) indicated strong agreement across domains (knowledge CVI = 0.91; self-efficacy CVI = 1.00; practice CVI = 1.00).

The knowledge component included factual statements about ACP and advance directives (ADs), with higher scores reflecting greater factual understanding; internal consistency was moderate (Cronbach’s α=0.58). The self-efficacy scale measured confidence in performing ACP-related tasks such as discussing prognosis, eliciting patient values, and facilitating completion of advance directive forms. A mean self-efficacy score was calculated across all items; internal consistency was high (Cronbach’s α=0.97). The practice domain comprised four scored items assessing ACP discussion, follow-up, discussion of palliative or hospice care, and discussion of appointing a surrogate decision-maker. Responses were coded 0 (‘No, I do not want to do it now nor in the future’), 1 (‘No, but I want to do it now or in the future’), or 2 (‘Yes’) and summed to a total practice engagement score from 0 to 8. Two conditional follow-up questions on the number and documentation of ACP discussions were descriptive and were not scored; internal consistency of the four-item practice scale was good (Cronbach’s α=0.83).

### Data collection

Surveys were distributed electronically and in paper format between December 2022 and May 2023. Respondents participated voluntarily and anonymously.

### Data analysis

We used IBM SPSS Statistics (version 22) for data analysis. Descriptive statistics were calculated for all variables. Demographic characteristics were summarised using means with standard deviations for continuous variables and frequencies with percentages for categorical variables. We computed the total knowledge score (0–11) and the mean self-efficacy score (1–10). For ACP practice, distributions were calculated for the four scored items and two conditional follow-up questions, and the four scored items were summed to form the 0–8 practice engagement score.

Associations between knowledge, self-efficacy, and the 0–8 ACP practice engagement score were examined using Pearson’s correlations, with the bounded practice score treated as approximately continuous. Independent associations were then estimated using hierarchical linear regression with the 0–8 practice engagement score as the outcome. Model 1 included education level, organisation affiliation, whether the organisation had implemented ACP, and prior end-of-life/ACP training; Model 2 added ACP knowledge and self-efficacy. Model assumptions (linearity, homoscedasticity, residual normality, and multicollinearity) were examined. We report unstandardised coefficients (B), standard errors, standardised coefficients, t values, 95% confidence intervals, and two-tailed p values (α=0.05).

### Ethical considerations

This study was approved by the Research Ethics Committee of [Anonymised for review] (Reference number: REC-2022.502). All participants provided informed consent before participation. Participation was voluntary, and participants could decline or withdraw without consequences. Data were kept confidential, stored securely, and accessed only by the research team. The study was conducted in accordance with the Declaration of Helsinki and applicable institutional requirements.

## Results

### Participant characteristics

A total of 213 HSCPs participated in the survey, including nurses (n = 143, 67.1%), physicians (n = 14, 6.6%), therapists (n = 16, 7.5%), and social workers (n = 40, 18.8%). The demographic profile of these participants is summarised in Table 1 below. The majority were female (n = 175, 82.2%), reflecting the gender distribution typical in nursing staff. Participants were categorized into three age groups. The majority were aged 31–45 years (48.8%), followed by those aged 30 years or below (40.4%), while only 10.8% were 46 years or above. Participants had a broad range of professional experience in their respective fields, with nearly half (49.3%) having worked for 6–15 years, followed by 29.6% with ≤5 years, 14.6% with 16–25 years, and 6.6% with ≥26 years of experience. Similarly, their tenure at their current institutions also varied, with 44.6% having worked ≤5 years, 42.7% for 6–15 years, 9.9% for 16–25 years, and only 2.8% for ≥26 years, indicating relatively high workforce mobility. Over half of the professionals reported having worked in tertiary hospital (n = 123, 57.7%), equal numbers of professionals worked in community day centres (n = 45, 21.1%) and community long-term care facilities (n = 45, 21.1%).

**Table 1:** Demographic characteristics of study respondents (n = 213).

Variables		n/ M	%/SD
Gender			
	Male	38	17.8
	Female	175	82.2
Age			
	≤30 years	86	40.4
	31–45years	104	48.8
	≥46 years	23	10.8
Education level			
	College/ Bachelor	145	68.1
	Postgraduate diploma	25	11.7
	Master’s or above	43	20.2
Profession			
	Nurse	143	67.1
	Physician	14	6.6
	Therapist	16	7.5
	Social worker	40	18.8
Professional experience (years)			
	≤5	63	29.6
	6–15	105	49.3
	16–25	31	14.6
	≥26	14	6.6
Year(s) working in current organisation			
	≤5	95	44.6
	6–15	91	42.7
	16–25	21	9.9
	≥26	6	2.8
Organisation category			
	Tertiary hospital	123	57.7
	Community day centre/ nursing day centre	45	21.1
	Community long-term care setting	45	21.1

### Advance care planning knowledge among professionals

Participants answered a mean of 72.8% of the 11 knowledge items correctly (Table 2). Correct-response rates ranged from 17.4% to 96.2%, indicating substantial variation across topics.

**Table 2:** Knowledge of ACP among professionals (n = 213).

Items	Correct rate %	True	False	Don’t know
1. ACP can be formulated at any time.	72.3	154	37	22
2. The purpose of ACP is to effectively communicate a patient's wishes for end-of-life care.	96.2	205	1	7
3. The best time to discuss ACP is when the patient is terminally ill.	83.1	27	177	9
4. ACP can only be activated when an individual is terminally ill or in an irreversible coma.	71.4	46	152	15
5. Once an ACP is established, it cannot be revoked or modified.	93.0	6	198	9
6. ACP allows patients to refuse life-sustaining treatments.	68.1	145	40	28
7. The implementation of ACP is a form of physician-assisted dying.	67.6	42	144	27
8. Only older individuals (aged 65 and above) can establish ACP.	89.2	6	190	17
9. A patient can designate more than one surrogate decision-maker.	17.4	117	37	59
10. A surrogate decision-maker can make decisions on behalf of a patient who still has decision-making capacity.	71.4	39	152	22
11. If a patient indicates in the ACP that they do not wish to receive life-sustaining treatments (such as ventilators, dialysis, etc.), even if the doctor disagrees with the patient's plan, they must still follow the patient's wishes.	71.4	152	34	27
Average correct rate	72.8			
Cronbach's alpha=0.58				

Note. Correct answers: 1 True; 2 True; 3 False; 4 False; 5 False; 6 True; 7 False; 8 False; 9 False; 10 False; 11 True.

Participants showed strong understanding of several fundamental concepts: 96.2% correctly identified that the purpose of ACP is to communicate patients' end-of-life care wishes, 93.0% recognised that an ACP can be modified or revoked, and 89.2% understood that ACP is not restricted to older adults.

The lowest correct-response rate was observed for item 9, concerning whether a patient may designate more than one surrogate decision-maker (17.4%). Knowledge gaps were also evident regarding the distinction between ACP and physician-assisted dying (item 7, 67.6% correct) and the use of ACP to refuse life-sustaining treatment (item 6, 68.1% correct). By contrast, 83.1% correctly recognised that ACP should not begin only in the terminal stage, and 71.4% correctly rejected the view that ACP applies exclusively to terminal illness or irreversible coma.

The item-level distribution therefore indicates that the overall mean should not be interpreted as uniform mastery of ACP's legal and procedural concepts.

### Self-Efficacy for advance care planning discussions

As shown in Table 3. below, professionals demonstrated moderately high self-efficacy in conducting ACP-related discussions, with an average item score of 5.7, Cronbach’s α = 0.97, indicating strong reliability.

**Table 3:** Self-efficacy for ACP discussions among professionals (n = 213).

Item	Mean Score
1. I am confident discussing issues related to ACP with patients.	5.9
2. I am confident discussing the refusal of life-sustaining treatments with patients (e.g., CPR, dialysis).	5.8
3. I am confident discussing future medical care with patients in the event they lose decision-making capacity (e.g., persistent vegetative state, advanced dementia).	5.9
4. I am confident managing patients’ negative emotions that arise during ACP discussions.	5.4
5. I am confident initiating ACP discussions with patients.	5.6
6. I am confident discussing the follow-up care involved in refusing or withdrawing life-sustaining treatments (e.g., palliative care, hospice care).	5.7
7. I am confident assisting patients in documenting ACP content in the medical record.	6.4
8. I am confident handling conflicts between patients and family members during ACP discussions.	5.2
9. I am confident knowing the appropriate timing to initiate ACP discussions with patients.	5.4
10. I am confident discussing issues related to dying or death with patients.	5.7
11. I am confident answering patients’ questions regarding their rights to refuse life-sustaining treatments.	5.6
Average score per item	5.7

Across individual items, participants reported the highest level of self-efficacy in assisting patients to document ACP content in medical records (Mean=6.4). They also expressed relatively high confidence in discussing general ACP topics (Mean=5.9), engaging in conversations about potential loss of decision-making capacity (Mean=5.9), and explaining the refusal of life-sustaining treatments (Mean=5.8–5.7).

However, lower levels of self-efficacy were observed in areas requiring interpersonal skills. Professionals felt less confident in handling patients’ negative emotions (Mean=5.4), initiating ACP discussions at the appropriate time (Mean=5.4), and especially in managing conflicts between patients and family members (Mean=5.2). These findings suggest that while professionals generally feel competent in factual and procedural aspects of ACP, they may require additional training and support in emotional communication and conflict resolution during ACP conversations.

### Engagement in advance care planning practice

There were two components in the investigation of ACP practices. The first examined whether participants’ organisations had incorporated ACP into their service systems and, if so, which professional groups were responsible for its implementation (Table 4). The second component explored participants’ actual engagement in ACP-related practices within their own work (Table 5).

**Table 4:** Implementation of ACP.

	n	%
Organisation has implemented ACP (n = 213)		
No	171	80.3
Yes	42	19.7
Professionals responsible for ACP in organisations (n = 42)		
Nurse	32	76.2
Physician	28	66.7
Physiotherapist	5	11.9
Occupational therapist	4	9.5
Social worker	14	33.3
Nutritionist	2	4.8

**Table 5:** ACP practice behaviours among professionals.

Variables		n	%
I have discussed ACP with terminally ill patients or their relatives			
	No, I do not want to do it now nor in the future	19	8.9
	No, but I want to do it now or in the future	164	77.0
	Yes	30	14.1
Documentation of ACP discussions in patient records (n = 30)			
	All discussions were documented	11	36.7
	Most discussions were documented	9	30.0
	Some discussions were documented	9	30.0
	No documentation	1	3.3
I have followed-up ACP with terminally ill patients			
	No, I do not want to do it now nor in the future	21	9.9
	No, but I want to do it now or in the future	161	75.6
	Yes	31	14.6
I have discussed palliative or hospice care with terminally ill patients or their relatives			
	No, I do not want to do it now nor in the future	19	8.9
	No, but I want to do it now or in the future	120	56.3
	Yes	74	34.7
I have discussed with terminally ill patients on appointing one surrogate decision-maker			
	No, I do not want to do it now nor in the future	22	10.3
	No, but I want to do it now or in the future	164	77.0
	Yes	27	12.7
Note. The first four items were scored 0 ('No, and I do not want to do it now or in the future'), 1 ('No, but I want to do it now or in the future'), or 2 ('Yes') and summed (range 0–8). The discussion-count and documentation questions were conditional follow-ups and were not scored. Cronbach's alpha for the four-item score=.83.			

As shown above, among the 213 professionals, only 19.7% (n = 42) reported that their organisations had implemented ACP, whereas the majority 80.3% (n = 171) indicated that ACP had not yet been incorporated into their workplace. Among the 42 participants whose organisations had implemented ACP, nurses were the most commonly responsible professionals (76.2%, n = 32), followed by physicians (66.7%, n = 28).

Presented in Table 5, a minority had engaged in ACP-related discussions or activities with terminally ill patients. Among all respondents, 14.1% reported that they had previously discussed ACP with terminally ill patients or their relatives, whereas the majority (77.0%) had not yet done so but expressed willingness to do so in the future.

Among the 30 participants who had conducted ACP discussions, the mean number of patients or relatives they had discussed ACP with was 13.5 (SD=27.2). Regarding documentation practices, just over one-third of participants (36.7%) reported documenting all ACP discussions in patient records. The remainder documented most discussions (30.0%), some discussions (30.0%), or no discussions (3.3%), indicating variability in documentation practices.

Follow-up of ACP with terminally ill patients was reported by 14.6% of participants, while 75.6% had not done so but were willing to engage in follow-up activities in the future. A further 9.9% indicated no intention to follow up ACP discussions either now or in the future.

Regarding palliative or hospice care discussions, 34.7% had discussed palliative or hospice care options with terminally ill patients or relatives, while 56.3% had not yet done so but intended to in the future. A small proportion (8.9%) did not intend to engage in such discussions. Finally, 12.7% reported having discussed the appointment of a surrogate decision-maker with terminally ill patients. The majority (77.0%) had not conducted such discussions but expressed willingness to do so in future practice, and 10.3% did not intend to carry out surrogate decision-maker discussions.

### Relationships between knowledge, self-efficacy, and practice

A central question of our study was how the professionals’ knowledge and self-efficacy relate to their engagement in ACP practices. Table 6. below displays the correlations among these variables.

**Table 6:** Correlations Between ACP Knowledge, Self-Efficacy, and ACP Practice Engagement.

**Correlation Analysis**			
	Knowledge	Self-efficacy	Practice
Knowledge	1		
Self-efficacy	0.09	1	
Practice	0.25***	0.33***	1
***p < 0.001			

Advance care planning knowledge was not significantly correlated with self-efficacy (r = 0.09), indicating that higher knowledge levels did not necessarily correspond to greater confidence in discussing or delivering ACP. In contrast, ACP practice engagement was significantly correlated with both knowledge and self-efficacy. Specifically, ACP practice showed a significant positive correlation with knowledge (r = 0.25, *p* < 0.001), suggesting that participants with greater ACP knowledge were more likely to engage in ACP-related behaviours. Additionally, ACP practice demonstrated a moderate positive correlation with self-efficacy (r = 0.33, *p* < 0.001), indicating that individuals who felt more confident in communicating about ACP were more actively involved in ACP practice.

### Factors associated with advance care planning practice behaviours

To identify factors associated with professionals’ engagement in ACP practice behaviours, hierarchical linear regression analyses were conducted using the 0–8 ACP practice engagement score as the dependent variable (Tables 7, 8). Model 1 examined sociodemographic and organisational factors, while Model 2 additionally included ACP knowledge and self-efficacy.

**Table 7:** Model 1 regression of ACP practice engagement scores (n = 213).

	B	SE B	beta	t	p	95% CI		VIF
Predictor						Lower Upper		
Constant	3.89	0.18		21.52	<.001	3.54	4.25	
Education (reference: bachelor's degree or below)								
Postgraduate diploma	0.42	0.35	.080	1.21	.227	−0.26	1.11	1.10
Master's degree	0.89	0.28	.211	3.19	.002	0.34	1.44	1.10
Organisation (reference: hospital/other)								
Community health centre	−0.51	0.28	-.124	−1.85	.066	−1.06	0.03	1.13
Long-term-care institution	0.22	0.28	.052	0.77	.440	−0.34	0.77	1.15
Organisation has implemented ACP (reference: no)								
Yes	0.95	0.28	.224	3.45	<.001	0.41	1.49	1.06
Prior ACP training (reference: no)								
Yes	0.69	0.28	.161	2.49	.014	0.14	1.24	1.05
Model 1: R=.423, R²=.179, adjusted R²=.155, F(6, 206)=7.49, p<.001

**Table 8:** Model 2 regression of ACP practice engagement scores (n = 213).

	B	SE B	beta	t	p	95% CI		VIF
Predictor						Lower	Upper	
Constant	1.62	0.56		2.91	.004	0.52	2.72	
Education (reference: bachelor's degree or below)								
Postgraduate diploma	0.35	0.34	.066	1.03	.304	−0.32	1.01	1.12
Master's degree	0.63	0.27	.150	2.32	.022	0.09	1.17	1.15
Organisation (reference: hospital/other)								
Community health centre	−0.53	0.27	-.129	−2.00	.046	−1.06	−0.01	1.13
Long-term-care institution	0.08	0.27	.019	0.29	.775	−0.46	0.61	1.18
Organisation has implemented ACP (reference: no)								
Yes	0.82	0.27	.192	3.06	.002	0.29	1.34	1.08
Prior ACP training (reference: no)								
Yes	0.33	0.28	.076	1.18	.240	−0.22	0.87	1.15
ACP knowledge score	0.13	0.06	.150	2.38	.018	0.02	0.24	1.08
Mean ACP self-efficacy score	0.25	0.06	.248	3.82	<.001	0.12	0.37	1.15
Model 2: R=.505, R²=.255, adjusted R²=.226, F(8, 204)=8.74, p<.001								

### Model 1: sociodemographic and organisational predictors

#### Educational level

Compared with participants holding a college or bachelor’s degree, those with a master’s degree or above reported significantly higher ACP practice engagement (B = 0.89, β=0.21, p = 0.002), see Table 7. This indicates that higher educational attainment was associated with more active involvement in ACP behaviours.

#### Organisational implementation of advance care planning

Professionals whose organisations had implemented ACP were significantly more likely to engage in ACP practice behaviours (B = 0.95, β=0.22, p < 0.001).

#### Previous advance care planning training

Receiving ACP-related training also significantly predicted higher levels of ACP practice

(B = 0.69, β=0.16, p = 0.014). This indicates that training was associated with ACP engagement in Model 1.

### Model 2: adding advance care planning knowledge and self-efficacy

Model 2 significantly improved model fit, explaining 25.5% of the variance (R²=0.255, adjusted R²=0.226, F(8, 204)=8.74, p < 0.001). When ACP knowledge and self-efficacy were included, both emerged as significant predictors; prior ACP training was no longer statistically significant (B = 0.33, p = 0.240).

### Advance care planning knowledge

Advance care planning knowledge was positively associated with ACP practice (B = 0.13, β=0.15, p = 0.018).

### Self-efficacy

Self-efficacy was a strong and significant predictor, with the largest psychological effect (B = 0.25, β=0.248, p < 0.001), indicating that professionals who felt more confident discussing ACP were more likely to engage in ACP practice.

### Organisation type

Working in community centres was negatively associated with ACP practice (B=−0.53, β=−0.129, p = 0.046), whereas working in hospitals served as the reference group.

## Discussion

This study examined the relationships between ACP knowledge, self-efficacy, and ACP practice among HSCPs in Macao. Consistent with our hypotheses, both ACP knowledge and self-efficacy emerged as significant correlates of ACP practice engagement. After controlling for demographic and organisational factors, professionals with higher ACP knowledge and greater self-efficacy were more likely to engage in ACP-related discussions and behaviours. These findings support Bandura’s social cognitive theory, which proposes that individuals are more likely to perform a behaviour when they believe they possess the capability to do so (Bandura, 1977; Bandura and Wessels1997). Within the context of ACP, professionals may possess sufficient factual knowledge about ACP, yet still hesitate to initiate conversations if they lack confidence in their communication skills, emotional management, or ability to navigate complex dynamics. Our findings therefore reinforce prior literature suggesting that confidence and preparedness are foundational to clinicians’ willingness to initiate ACP conversations (Suen et al., 2024; Zhu et al., 2023).

Despite the recognised importance of ACP for improving end-of-life communication and decision-making, this study revealed low levels of ACP engagement among HSCPs. This finding suggests that barriers to ACP implementation may extend beyond knowledge and confidence alone and may also reflect personal beliefs, perceived professional role boundaries, organisational culture, or broader sociocultural attitudes towards end-of-life discussions. Future qualitative research is warranted to explore these barriers in greater depth. This pattern is consistent with recent evidence describing limited ACP practice and multilevel implementation barriers in Asian and other healthcare settings (Cai et al., 2023; Inokuchi et al., 2023; Zhu et al., 2023).

Although nurses comprised over two-thirds of the study sample, this study included a multidisciplinary group of health and social care professionals, reflecting the collaborative nature of ACP implementation across healthcare settings. Nurses, who often spend considerable time with patients and families throughout the care continuum, are well positioned to initiate ACP discussions and facilitate ongoing communication about future care preferences. These findings reinforce the importance of viewing ACP as an interdisciplinary process rather than one led exclusively by physicians. Strengthening ACP competencies across nurses and other HSCPs may therefore enhance the integration of ACP into routine clinical practice.

Other factors may contribute to clinicians’ reluctance to initiate ACP. Advance care planning requires professionals to support patients in articulating values, preferences, and goals of care, areas deeply shaped by cultural norms, family dynamics, and societal expectations about illness and dying (McDermott & Selman, 2018; Suen et al., 2024). In Chinese-speaking contexts, where explicit discussions about death are traditionally discouraged, clinicians may feel uncomfortable initiating ACP or may anticipate resistance from patients and families (Chen et al., 2017; Lei et al., 2022). As such, cultural sensitivities may amplify emotional, communicative, and ethical uncertainties that reduce professionals’ willingness to begin ACP discussions.

Compared with HSCPs working in hospitals, those based in community centres for older adults were significantly less likely to engage in ACP-related behaviours. This finding contrasts with much of the existing literature, where studies on ACP promotion and practice are commonly situated in community care settings, and community-based professionals are often positioned as key facilitators of ACP (Garden et al., 2022; Ohr et al., 2021; Yeung et al., 2023). In our study, however, hospital-based professionals demonstrated higher levels of ACP engagement.

One possible explanation is that community organisations in Macao primarily provide recreational and wellness services for relatively healthy older adults, resulting in fewer opportunities to initiate discussions about future care planning. In contrast, hospital professionals more frequently encounter treatment decisions such as Do-Not-Attempt-Resuscitation orders, which may increase both the perceived relevance and urgency of ACP discussions. These findings suggest that both organisational context and individual capability influence professionals’ engagement in ACP practice.

Prior ACP training was significantly associated with ACP practice engagement in Model 1, but the association was attenuated and was not statistically significant after knowledge and self-efficacy were entered in Model 2 (B = 0.33, p = 0.240). This pattern is consistent with—but does not demonstrate—the possibility that training influences practice partly through knowledge or self-efficacy. No formal mediation analysis was conducted, and the cross-sectional design cannot establish temporal order. Organisational implementation of ACP remained significantly associated with practice engagement. Training programmes that incorporate role-play, simulation, reflective practice, and supervised ACP conversations may nevertheless strengthen professionals’ confidence by providing opportunities to practise communication skills in a supportive environment. ACP education should therefore extend beyond knowledge transfer to include experiential learning, while being supported by organisational implementation processes.

Despite the low level of actual ACP practice, most professionals expressed willingness to undertake ACP discussions in the future. This positive attitude represents an important facilitator for future ACP implementation, suggesting that the challenge lies not in resistance to ACP itself but in equipping professionals with the confidence, communication skills, and organisational support needed to translate willingness into practice.

These findings indicate that system-level support, including formal training, organisational policy, and structural integration of ACP processes, can enhance professionals’ engagement in ACP (Chan et al., 2023; Inokuchi et al., 2023; Shih et al., 2025). Our findings underscore the need for multi-level strategies to improve ACP practice among HSCPs in Macao. Interventions that combine knowledge acquisition with experiential learning, including simulation-based training and structured communication tools, may strengthen professionals’ self-efficacy, reduce discomfort in ACP conversations, and increase their readiness to initiate ACP (Ellis et al., 2021; Millstein et al., 2022). Such strategies may facilitate the translation of ACP knowledge into confident clinical practice and ultimately promote earlier, person-centred advance care planning in Macao.

## Limitations

This study has several limitations. First, the relatively small sample size may limit the generalisability of the findings to all HSCPs in Macao. Second, the use of self-administered questionnaire may have introduced selection bias, as professionals with greater interest in ACP may have been more likely to participant. Self-reported questionnaire are also susceptible to social desirability bias and recall bias, and participants may not have recognised informal discussions about future care planning as ACP, potentially underestimating actual ACP practice. The cross-sectional design precludes causal inference regarding the relationship among ACP knowledge, self-efficacy, and practice behaviours.

## Implications

The current study highlighted the need for ACP education that goes beyond knowledge transfer. Training for HSCPs should prioritise practical communication skills and confidence-building. Future research could evaluate whether ACP training and organisational implementation improve not only professional outcomes, such as knowledge, self-efficacy, and practice behaviour, but also patient- and family-centred outcomes, including care preference documentation, decision-making preparedness, and quality of end-of-life care.

## Conclusion

This cross-sectional survey provides crucial baseline evidence regarding ACP knowledge, self-efficacy, and practice among HSCPs in Macao. Although participants demonstrated moderate knowledge of ACP, their engagement in ACP practice remained limited. ACP knowledge and self-efficacy were both associated with ACP practice, self-efficacy showing the stronger relationship. These findings suggest that improving ACP implementation in Macao requires not only knowledge-based education, but also training that strengthen professionals’ confidence and communication skills in initiating ACP discussions.

## Funding information

The study was funded by the Kiang Wu Nursing College of Macao. The first author was funded via a postdoctoral programme by the Science and Technology Development Fund of Macao.

## CRediT authorship contribution statement

**Kuai In Tam:** Writing – review & editing, Writing – original draft, Methodology, Formal analysis, Conceptualization. **Sok Leng Che:** Writing – review & editing, Methodology, Formal analysis, Conceptualization. **Sok Man Leong:** Writing – review & editing, Methodology, Formal analysis, Conceptualization.

## Declaration of competing interest

The authors declare that they have no known competing financial or personal interests that could have appeared to influence the work reported in this paper.
