# Supplementary material for: Knowledge and self-efficacy in starting advance care planning among community health and social care professionals in macao: A cross-sectional study
Source: Int J Nurs Stud Adv. 2026 Oct 18;11:100716. doi: 10.1016/j.ijnsa.2026.100716 (PMC13718641; doi:10.1016/j.ijnsa.2026.100716)
Supplement: Supplementary file 1 [file mmc1.docx]

**澳門醫療及社會服務專業人員對預設照顧計畫之認知、信心和行為**

**調查問卷**

您好！

非常感謝您參加澳門鏡湖護理學院的研究！

本研究的調查對象為澳門醫療及社會服務專業人員。調查目的是為瞭解醫療及社會服務專業人員對預設照顧計畫之認知，以及在工作中開展預設照顧計劃的信心和行為，研究結果是相關服務發展的重要參考依據。

此份調查問卷填寫約需時5~10分鐘，採匿名自願參與的方式，您的私隱完全受到保障，研究結果將以集體的方式呈現，個人化資訊將不會對外公開。如在填寫問卷過程中有任何情感或私隱因素而不便繼續回答，有權隨時退出研究。

請問您是否在澳門具有醫療人員或社工的執業資格？

□ 是。（繼續回答）

□ 否。（結束問卷）

請問您是否於醫院、院舍、日間護理中心或長者中心中工作？

□ 是。（繼續回答）

□ 否。（結束問卷）

現請回答以下的問題以確認您了解以上所述，並勾選您的參與意願。

□ 我已清楚明白以上所述，願意參與調查。（開始問卷）

□ 不便參與，謝謝。（結束問卷）

若對調查有任何疑問，參與者可在調查前或調查中於辦公時間內與本研究小組聯絡：[Anonymised for review]。

調查完成後，若有任何和調查相關的問題，請與本研究小組聯絡。項目負責人：譚瑰賢講師；電話：[Anonymised for review]；電郵：[Anonymised for review] 。

如您對參與本調查有任何投訴或意見，請致電澳門鏡湖護理學院科研管理暨發展部（電話：[Anonymised for review]）。

**一、有關預設照顧計劃的知識**

請判斷以下內容的對錯。

| **題目** | **對** | **錯** | **不知道** |
| --- | --- | --- | --- |
| 1. 預設照顧計劃可在任何時候制定。 |  |  |  |
| 2. 預設照顧計劃的目的是為能有效地與病人溝通其對於臨終照護的願望。 |  |  |  |
| 3. 討論預設照顧計劃的最好的時機是當病人病情嚴重時。 |  |  |  |
| 4. 只有個人身患絕症或處於不可逆轉的昏迷狀態時才能啟動預設照顧計劃。 |  |  |  |
| 5. 一旦制定預設照顧計劃，則不能撤銷或修改。 |  |  |  |
| 6. 預設照顧計劃允許病人拒絕維持生命的治療。 |  |  |  |
| 7. 預設照顧計劃的實施是醫生協助病人死亡的一種形式。 |  |  |  |
| 8. 只有老年人（65歲及以上）才能制定預設照顧計劃。 |  |  |  |
| 9. 一個病人可指定一個以上的監護人。 |  |  |  |
| 10. 一個監護人可以為一個仍具有決定能力的病人做決定。 |  |  |  |
| 11. 如果病人在預設照顧計劃中說明不接受維生治療（如呼吸機、透析治療等），即使醫生不同意病人的計劃，仍需按照病人的意願執行。 |  |  |  |

Adapted from:

Chen, Y., Cheng, Q., Wang, Y., Liu, X., Li, X., Mao, T., & Peng, J. (2019). Knowledge-attitude-practice and counter-measures of Advance Care Planning among nurses. *Chinese Nursing Management 19*(3), 334-340.

**二、預設照顧計劃溝通自信量表**

請按您自己對於執行以下預設照顧計畫相關事情的信心評分。評分範圍為1至10分，1分為完全沒有信心，10分為非常有信心。

| **題目** | **1-10分** |
| --- | --- |
| 1. 我有信心與病人討論有關預設照顧計劃的議題。 |  |
| 2. 我有信心與病人討論拒絕維持生命治療的議題（例如：心肺復甦術、血液透析等）。 |  |
| 3. 我有信心與病人討論未來萬一他／她面臨失去自主能力時（例如：植物人、極重度失智…等）的醫療照護。 |  |
| 4. 我有信心處理病人在討論預設照顧計劃過程中所產生的負面情緒。 |  |
| 5. 我有信心能主動開始與病人討論預設照顧計劃的議題。 |  |
| 6. 我有信心能與病人討論拒絕或撤除維持生命治療的後續照顧（例如：紓緩治療／安寧照護等）。 |  |
| 7. 我有信心協助病人將預設照顧計劃的內容記錄在病歷中。 |  |
| 8. 我有信心能處理病人與家屬在討論預設照顧計劃過程中所發生的衝突。 |  |
| 9. 我有信心要在什麼時機點切入與病人討論預設照顧計劃的議題。 |  |
| 10. 我有信心與病人討論有關瀕死或死亡的議題。 |  |
| 11. 我有信心回答病人有關拒絕維持生命治療權利的相關問題。 |  |

Adapted from:

Chen, Y., Cheng, Q., Wang, Y., Liu, X., Li, X., Mao, T., & Peng, J. (2019). Knowledge-attitude-practice and counter-measures of Advance Care Planning among nurses. *Chinese Nursing Management 19*(3), 334-340.

Hsieh, C.-C., Huang, H.-P., Tung, T.-H., Chen, I. C., Beaton, R. D., & Jane, S.-W. (2019). The exploration of the knowledge, attitudes and practice behaviors of advanced care planning and its related predictors among Taiwanese nurses. *BMC Palliative Care*, *18*(1), 99. https://doi.org/10.1186/s12904-019-0483-9

**三、有關預設照顧計劃討論中的實踐行為**

在**過去6個月**，您是否曾完成以下事情？

| 1. 您是否與疾病末期病人或家屬討論過預設照顧計劃？ | | | | |
| --- | --- | --- | --- | --- |
|  | □是（續答第1a及1b題） | | | |
|  | □否，但是現在或將來想做（跳答至第2題） | | | |
|  | □否，現在或將來都不想做（跳答至第2題） | | | |
|  | 1a. 您和多少位病人（或其家屬）討論過？ | | | 位 |
|  | 1b. 是否在病人的檔案中記錄預設照顧計劃的討論結果？ | | | |
|  | □全部都有記錄　　□大部有記錄　　　□小部有記錄　　□沒有記錄 | | | |
| 2. 您是否有跟進疾病末期病人的預設照顧計劃？ | | | | |
|  | □是 | □否，但是現在或將來想做 | □否，現在或將來都不想做 | |
| 3. 您是否有與末期病人／家屬談論關於紓緩治療／安寧照護的選擇？ | | | | |
|  | □是 | □否，但是現在或將來想做 | □否，現在或將來都不想做 | |
| 4. 您是否與末期病人討論過指定一位能為自己作出治療選擇的監護人？ | | | | |
|  | □是 | □否，但是現在或將來想做 | □否，現在或將來都不想做 | |

Adapted from:

Chen, B., Fan, V. Y., Chou, Y. J., & Kuo, C. C. (2017). Costs of care at the end of life among elderly patients with chronic kidney disease: patterns and predictors in a nationwide cohort study. *BMC Nephrol*, *18*(1), 36. https://doi.org/10.1186/s12882-017-0456-2

**四、基本資料**（請於您認為最適當答案之空格中打勾）

| 1. 您的性別： | | | □男 | | | | | | | | | | | □女 | | | | | | |
| --- | --- | --- | --- | --- | --- | --- | --- | --- | --- | --- | --- | --- | --- | --- | --- | --- | --- | --- | --- | --- |
| 2. 您的年齡： | | | 歲（足歲） | | | | | | | | | | | | | | | | | |
| 3. 您的最高教育程度： | | | | | □大專 | | | | □本科／學　士 | | | | | □碩士或以上 | | | | | | |
|  | | | | | □其它： | | | | | | | | | | | | | | | |
| 4. 您目前的婚姻狀況： | | | | | □未婚 | | | | | | | | | | □已婚／同居 | | | | | |
|  | | | | | □分居／離婚 | | | | | | | | | □喪偶 | | | | | | |
| 5. 你目前的子女數目為（包括領養子女，但不包括契仔女）： | | | | | | | | | | | | | | | | | | | | 個 |
| 6. 您有沒有宗教信仰？ | | | | | | □沒有（跳答至第7題） | | | | | | | | | | | | | | |
|  | | | | | | □有（繼續回答6.1題） | | | | | | | | | | | | | | |
|  | 6.1 您的宗教信仰為： | | | | | □天主教 | | | | | | | □基督教 | | | | | | □佛教 | |
|  | | | | | | □民間信仰（如拜神） | | | | | | | | | | | | □其它： | | |
| 7. 您的專業為： | | | | | | □護士 | | | | | | | □醫生 | | | | | | □物理治療師 | |
|  | | | | | | □職業治療師 | | | | | | | □社工　　　　□其他： | | | | | | | |
| 8. 從事此專業的年資為？ | | | | | | 年 | | | | | | | | | | | | | | |
| 9. 在目前機構工作時間為？ | | | | | | 年 | | | | | | | | | | | | | | |
| 10. 您的工作機構為？ | | | | | | □醫院 | | | | | | | □院舍 | | | | | | □日間護理中心 | |
|  | | | | | | □長者中心 | | | | | | | □其他： ________ | | | | | | | |
| 11. 過去一個月平均每天服務個案數量為？ | | | | | | 個 | | | | | | | | | | | | | | |
| 12. 您工作的機構目前是否有開展預設照顧計劃？ | | | | | | | | | | | | | | | | | | | | |
|  | □有（繼續回答12.1題） | | | | | | | | | □沒有（跳答第13題） | | | | | | | | | | |
|  | 12.1您工作的機構中主要由哪種專業人員開展預設照顧計劃？ | | | | | | | | | | | | | | | | | | | |
|  | | □護士 | | □醫生 | | | | □物理治療師 | | | | □職業治療師 | | | | | □社工　　　　　□其他： | | | |
| 13. 您過去是否曾接受預設照顧計劃培訓？ | | | | | | | | | | | | | | | | | | | | |
|  | □是（繼續回答13.1題） | | | | | | | | | | □否（完成問卷） | | | | | | | | | |
|  | 13.1 您曾接受的預設照顧計劃培訓形式為何？（可多選） | | | | | | | | | | | | | | | | | | | |
|  | | □講座 | | | | | □工作坊 | | | | | | | | | □證書課程 | | | | |

**》》》》》》》》》》》問卷結束，謝謝您的回答《《《《《《《《《《《**
